# Circulating endothelial progenitor cells

**DOI:** 10.1038/sj.bjc.6602808

**Published:** 2005-09-27

**Authors:** B Garmy-Susini, J A Varner

**Affiliations:** 1 John and Rebecca Moores Comprehensive Cancer Center, University of California, San Diego, 3855 Health Sciences Drive, La Jolla, CA 92093-0819, USA

**Keywords:** angiogenesis, stem cell, endothelial progenitor cell, CD34+ cell, monocyte

## Abstract

Angiogenesis research investigates the formation of new blood vessels in wound healing, tumour growth and embryonic development. Circulating, bone marrow-derived endothelial progenitor cells (EPCs) were first described 8 years ago, yet the exact nature of these endothelial precursor cells remains unclear. The contributions of circulating EPCs to angiogenesis in tumours, ischaemic injury and other diseases as well as their usefulness in the repair of wounded hearts and limbs remain under intense investigation.

The growth of new blood vessels, or neovascularisation, is required for embryonic development and stimulates the healing of injured tissues, but also promotes tumour growth and inflammatory diseases. Quiescent vascular and lymphatic endothelial cells are activated by proangiogenic growth factors such as vascular endothelial growth factor (VEGF), which stimulate endothelial cell proliferation and migration, thereby promoting new vessel formation (Stacker *et al*, 2002; Jin and Varner, 2004). Thirty years of analysis has revealed many of the mechanisms driving blood vessel sprouting. However, in 1997, the study of vascular development was changed when Dr Jeffrey Isner and his research team at Tufts University first described circulating, bone marrow-derived endothelial progenitor cells (EPCs) (Asahara *et al*, 1997). In the 8 years since, mounting evidence has shown that endothelial cells can arise from circulating bone marrow-derived cells. Human bone marrow-derived cells have been shown to infiltrate human tumours (Peters *et al*, 2005) and to give rise to up to 16% of the tumour neovasculature in a normal mouse (Ruzinova *et al*, 2003). However, controversies about the phenotype of this circulating progenitor cell have not completely resolved which circulating cells give rise to endothelial cells during *in vivo* angiogenesis (Figure 1).

## INITIAL CHARACTERISATION OF EPCS

Circulating EPCs were first identified as a population of postnatal mononuclear blood cells that adopted an adherent, endothelial morphology when cultured for 7 days in endothelial growth medium (Asahara *et al*, 1997). These initial studies showed that a CD34-enriched (15% CD34+) subpopulation of mononuclear blood cells formed blood island-like colonies on fibronectin-coated culture plates. CD34 is a marker of haematopoietic stem cells and is expressed by 0.1% of circulating mononuclear cells. Like endothelial cells, these isolated cells incorporated acetylated DiI, expressed several markers in common with endothelial cells such as CD34, CD31, vascular endothelial growth factor receptor 2 (VEGFR2) and Tie2. A small percentage of labelled human EPCs were able to incorporate into blood vessels in athymic nude mice in which hindlimb ischaemia was induced *in vivo*. Murine *β*-galactosidase+EPCs and rabbit EPCs also incorporated into blood vessels in ischaemic tissue (Asahara *et al*, 1997). Further studies in mice transplanted with *β*-galactosidase+(LacZ+) bone marrow showed clearly that bone marrow-derived cells naturally participate in the formation of new blood vessels in ischaemic limb (Asahara *et al*, 1999) and ischaemic heart. These transplanted cells improved blood flow in animals with hindlimb ischaemia. Further analysis of these cells indicated that they expressed not only the endothelial cell markers CD34, CD31, VEGFR2 and VE-cadherin but also the myeloid cell marker CD14 (Kalka *et al*, 2000).

## FUNCTIONAL EVIDENCE FOR THE BONE MARROW COMPONENT OF ADULT ANGIOGENESIS

Striking evidence supporting the existence of a bone marrow-derived component of angiogenesis arose from studies of mice with mutations in the Id family of transcriptional repressors. Id1+/−Id3−/− mice exhibited defects in postnatal angiogenic sprouting and suppressed tumour growth (Lyden *et al*, 2001). However, tumours grew normally in Id mutant mice transplanted with bone marrow from the wild-type mice. The vasculature of 100% of tumours grown in Id mutant mice transplanted with Rosa 26 (LacZ+) bone marrow was LacZ+, demonstrating that much of the vasculature originated from bone marrow (Lyden *et al*, 2001). The importance of Id genes in tumour angiogenesis was also observed in PTEN+/− mice, which exhibit spontaneous lymphomas, uterine carcinomas, prostate intraepithelial neoplasias and pheochromocytomas. Tumour angiogenesis and growth, but not incidence, was suppressed in PTEN+/−Id1+/−Id3−/− mice (Ruzinova *et al*, 2003). Transplantation of PTEN+/−Id1+/−Id3−/− mice with Rosa 26 bone marrow promoted tumour angiogenesis and indicated that different tumours recruited bone marrow-derived endothelial cells at different rates. While 16% of the vasculature in uterine carcinomas derived from the bone marrow, none of the vasculature in lymphomas did, suggesting that different tumours recruit bone marrow-derived cells to different degrees (Ruzinova *et al*, 2003).

Additional evidence supporting the concept of bone marrow-derived EPCs has been garnered from mouse bone marrow transplantation models. In mice, bone marrow-derived progenitor cells are c-kit+ cells that are deficient in expression of lineage markers (Lin−). For example, GFP+Lin− progenitor cells isolated from GFP+ mice have given rise to GFP+ retinal blood vessels when injected intravitrally into neonatal mice (Otani *et al*, 2002). Importantly, transplantation of single Lin-Sca1+GFP+ bone marrow-derived cells into mice not only generated GFP+ cells of all haematopoietic lineages but also GFP+ endothelial cells in angiogenic tissues (Grant *et al*, 2002).

More evidence for the bone marrow-derived EPC has arisen from studies on human CD34+ progenitor cells. Bone marrow-derived CD34+ progenitor cells include haematopoietic short- and long-term repopulating stem cells and partially committed progenitor cells of the myeloid/erythroid and lymphoid lineages. While CD34+ cells comprise a small percentage of the total circulating white blood cell population (0.1%), angiogenic growth factors and other chemokines such as GM-CSF induce significant release of these cells from the bone marrow into the circulation. Isolation of these cells by immune selection techniques has permitted a number of studies that have shown that purified CD34+ progenitor cells can promote angiogenesis and stimulate tissue repair *in vivo*. In one key study, CD34+ stem cells were used to promote neurogenesis after stroke in animal models. Surprisingly, rather than directly stimulating neurogenesis, CD34+ cells promoted angiogenesis, indirectly improving neuronal function (Taguchi *et al*, 2004).

Analyses of human sex-mismatched bone marrow transplantation patients provided evidence that endothelial cells do arise from bone marrow in humans. In one study, a small percentage of vasculature of sex-mismatched transplant patients was derived from the transplanted bone marrow. When patients were analysed on average 1 year after transplantation, 2% of all endothelial arose from the donor bone marrow (Jiang *et al*, 2004). In another study of human sex-mismatched bone marrow transplant recipients who later developed tumours, fluorescence *in situ* hybridisation analysis showed that approximately 5% of endothelial cells infiltrating tumours were derived from bone marrow (Peters *et al*, 2005). Thus, experimental and clinical data confirm the existence of bone marrow-derived endothelial progenitors.

## RELATIONSHIP TO DEVELOPMENTAL ANGIOGENESIS

A common precursor for haematopoietic and endothelial cells, the hemangioblast, was first described by developmental biologists. Endothelial cells and haematopoietic cells both arise in the blood islands of the yolk sac, in the ventral wall of the aorta and in the aorta–gonal–mesonephros region of the embryo. *In vitro* studies of embryoid body formation from embryonic stem cells show that both endothelial cells and haematopoietic cells arise from Flk-1+ (VEGFR2) colonies (Choi *et al*, 1998). Additionally, embryonic deletion in mouse of the transcription factor Scl/Tal1 suppresses haematopoiesis, while knockdown of Scl in zebrafish inhibits vascular development (Patterson *et al*, 2005). Mice deficient in the AML-1 gene exhibit defective haematopoiesis and embryonic lethality. *In vitro* studies of para-aortic splanchnopleural explants showed that AML-1-deficient mice also exhibit defective vascular development (Takakura *et al*, 2000). Together with evidence that developing endothelial cells and haematopoietic cells arise from a single common precursor CD31+VEGFR2+VE-cadherin+CD45− (Wang *et al*, 2004), these studies indicate that endothelial cells and haematopoietic cells arise from a common precursor. Importantly, embryonic EPCs (c-kit+Sca1+Flk^low^Tie2+) have been shown to give rise to tumour vasculature when injected into adult mice undergoing tumorigenesis (Vajkoczy *et al*, 2003).

In postnatal animals, proliferating endothelial cells and haematopoietic progenitor cells also share certain surface determinants. Both are CD34+VEGFR2+ cells that also express integrins *α*4*β*1 and *α*5*β*1. In fact, transplantation of a single GFP+ haematopoietic precursor cell in mice resulted in mice with GFP+ haematopoietic cells and GFP+ endothelial cells in angiogenic tissues (Grant *et al*, 2002). These studies suggest that postnatal hemangioblasts not only exist but can also be isolated and used to repopulate either endothelial or haematopoietic lineages.

## CHARACTERISATION OF EPCS BY MARKER ANALYSIS

Many recent studies support the existence of a postnatal bone marrow-derived endothelial precursor cell, the hemangioblast. Yet, the exact nature of this cell type remains unclear. The first EPCs were described as CD34-enriched mononuclear cells that acquired endothelial surface marker expression in culture (Asahara *et al*, 1997). Subsequent studies showed that a subpopulation of circulating CD34+ cells expressing CD34+CD133+VEGFR2+ could form endothelial colonies *in vitro* (Peichev *et al*, 2000; Gill *et al*, 2001). Yet, other studies have shown that CD11b+ or CD14+ mononuclear cells give rise to endothelial cell-like colonies *in vitro* or *in vivo* (Fujiyama *et al*, 2003; Gulati *et al*, 2003; Rehman *et al*, 2003; Yang *et al*, 2004; Chavakis *et al*, 2005; Urbich *et al*, 2005). Thus, it appears that endothelial-like cells can arise from within bone marrow-derived haematopoietic progenitor cell populations (CD34+ cells) or monocyte populations (CD14+ or CD11b+ cells). The actual identity of the endothelial precursor may cross the boundaries of the stem cell and monocyte populations (Schmeisser *et al*, 2001). A recent study showed that CD34+ CD11b+ cells from cord blood yielded endothelial colonies (Hildbrand *et al*, 2004). Thus, human endothelial cell precursors may express both progenitor cell markers (CD34, CD133), monocyte markers (CD14 and or CD11b) and endothelial markers (VEGFR2). Interestingly, gene expression analysis comparing EPCs to endothelial cells indicated that human endothelial cell precursors closely resemble freshly isolated endothelial cells from tumours rather than cultured endothelial cells (Bagley *et al*, 2003).

In studies from mice, endothelial precursor cells have derived from Lin− cells or from Lin-Sca1+c-kit+ subpopulation of bone marrow mononuclear cells. Some variability has been observed in the *in vivo* contribution of EPCs to angiogenesis in mice, leading to scepticism about the importance of this aspect of angiogenesis. However, Shaked *et al* (2005) recently showed that different strains of mice exhibit vastly different levels of circulating EPCs. These results suggest that some of the different estimates of EPC contributions to angiogenesis may be due to intrinsic strain or tissue recruitment capabilities.

Many studies on the roles of bone marrow-derived angiogenesis have been performed in FVB/N mice transplanted with Tie2LacZ bone marrow (Asahara *et al*, 1997, 1999; Lyden *et al*, 2001). Tie2 is best known as an endothelial cell marker; however, this protein can be expressed by monocytes that may contribute to angiogenesis by secreting growth factors. In fact, at least one recent study suggested that many of the Tie2 expressing bone marrow-derived cells in angiogenic tissues are not endothelial precursor cells but rather F4/80, CD11b-positive monocytes (de Palma *et al*, 2003). However, these studies differed from previous studies by transfecting bone marrow-derived progenitor cells with lentiviral vectors expressing GFP under control of a Tie2 promoter. It is possible that differences in mouse strains or technical methodology accounts for the absence of Tie2-positive endothelial cells in the de Palma studies.

Some of the variability in the characterisation of EPCs may arise as a result of different purification methods. Originally, EPCS were isolated by enriching for CD34+ cells and culturing in endothelial growth medium on fibronectin-coated substrates (Asahara *et al*, 1997). Further studies with CD34+ cells purified by immunoaffinity chromatography showed that these cells had long-term potential to form endothelial cell-like colonies *in vitro*. However, some EPCs have been isolated by culturing bone marrow-derived mononuclear cells on fibronectin-coated plates for several days. These cells form blood island-like colonies *in vitro* and some of these CD34− populations have been shown to give rise to EPCs. Continued pursuit of the identity of the EPC and study of its differentiation and longevity will clarify the current discrepancies within the field.

## REGULATION OF EPC RECRUITMENT

A number of growth factors and chemokines promote postnatal angiogenesis. Best characterised of these is VEGF. During tumour angiogenesis or after ischaemic injury, the level of circulating VEGF have been shown to rise (Senger *et al*, 1986; Connolly *et al*, 1989; Leung *et al*, 1989). This increased circulating VEGF promotes the mobilisation of EPCs from the bone marrow (Asahara *et al*, 1999; Hattori *et al*, 2001). In contrast, Ang-1 inhibited EPC release from bone marrow (Hattori *et al*, 2001). Most recently, the novel growth factor PDGF-CC was shown to promote revascularisation of ischaemic tissues in part by stimulating outgrowth of CD34+CD133+ EPCs (Li *et al*, 2005).

## THERAPEUTIC AND PROGNOSTIC POTENTIAL

One of the greatest hopes for the study of endothelial progenitors is their potential use in the therapy of ischaemic disease (Jain and Duda, 2003). Soon after their discovery, endothelial progenitors were used in attempts to restore blood flow to animals with hindlimb ischaemia (Asahara *et al*, 1997, 1999). In these studies, adoptive transfer of EPCs promoted restored blood flow and increased capillary density, resulting in decreased loss of limbs (Kalka *et al*, 2000). Further studies showed that introduction of EPCs by intracardiac catheter promoted recovery from myocardial ischaemia (Kawamoto *et al*, 2002). On the basis of these and other similar studies, clinical pilot studies were performed to investigate the use of EPCs in the restoration of blood flow in patients with hearts that have been damaged by ischaemia (reviewed by Losordo and Dimmeler, 2004). In the Transplantation of Progenitor Cells and Regeneration Enhancement in Acute Myocardial Infarction (TOPCARE-AMI) trial, patients received infusions of bone marrow-derived mononuclear cells or EPCs approximately 4 days after myocardial infarction. Patients receiving either population of cells exhibited improved ejection fraction and end-systolic volumes, indicating improved cardiac function. Several other clinical studies showed similar effects (reviewed by Losordo and Dimmeler, 2004).

Recent studies suggest that enumeration of circulating EPCs in patients may be useful in predicting the outcome of therapy or disease course (Bertolini *et al*, 2003; Schmidt-Lucke *et al*, 2005). Studies in mice indicate that the number of circulating EPCs is affected by systemic exposure to angiogenic regulators such as VEGF and can decline in response to antiangiogenic therapy such as anti-VEGFR2 antibody therapy (Shaked *et al*, 2005). Chemotherapeutic drug response can be measured in part by the level of EPCs in circulation (Bertolini *et al*, 2003). Thus, measurement of circulating EPCs may provide a useful assessment of disease susceptibility or to response to therapies.

## CONCLUSIONS

The field of angiogenesis research was revolutionised 8 years ago, with the discovery of the circulating EPC. Since then, the nature of this cell type and its abilities to form endothelial cell colonies *in vitro* and *in vivo* have been studied. *In vivo* clinical studies have been initiated to determine the potential of these cells to stimulate tissue repair after ischaemic injury. While the exact subpopulation of bone marrow-derived cells that can give rise to endothelial colonies has not been identified, studies suggest that CD34+CD11b+ cells contain these precursor cells. Improved purification of these progenitor cells and continued study of their long-term potential to generate endothelial cells *in vivo* will clarify this embryonic field of vascular research.

## Figures and Tables

**Figure 1:**
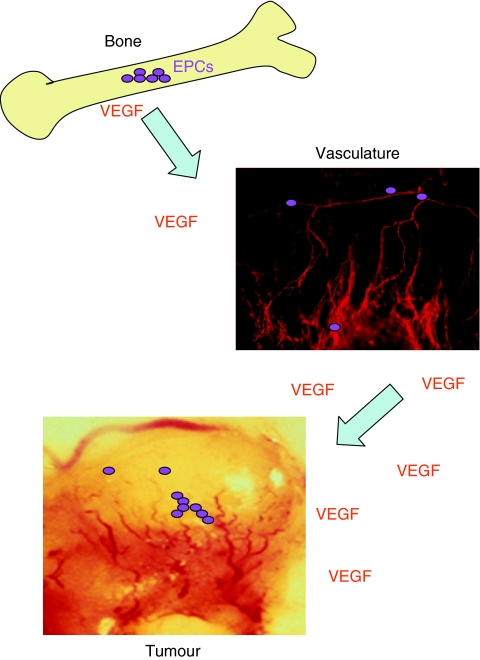
Bone marrow-derived endothelial progenitor cells (EPCs) contribute to tumour angiogenesis. Endothelial progenitor cells (purple circles) arise in the bone marrow as CD34+CD133+VEGFR2+ cells. These cells are induced to leave the bone marrow and enter the vasculature by circulating angiogenic factors such as vascular endothelial growth factor (VEGF). Once in the circulation, these cells can arrest at sites of ischaemia or growth factor release (such as VEGF release), such as in the tumour periphery. These cells then can participate in new vessel formation by differentiating into branching blood vessels.
